# Pediatric Elbow Dislocation With Comminuted Fractures of the Medial Humeral Epicondyle and Trochlea

**DOI:** 10.7759/cureus.110442

**Published:** 2026-06-08

**Authors:** Tanzeelur N Rahman, Giriraj Harshavardhan, Sundar Suriyakumar, Mohammed Tavfiq

**Affiliations:** 1 Department of Orthopaedics, Sri Ramachandra Institute of Higher Education and Research, Chennai, IND

**Keywords:** conservative management, elbow dislocation, medial epicondyle fracture, pediatric fracture, trochlear fracture

## Abstract

Elbow dislocations in children are uncommon and often occur alongside fractures, most frequently of the medial epicondyle. Concurrent dislocation with fractures of both the medial epicondyle and the trochlea is exceptionally rare. We present an unusual case of an 11‑year‑old boy with elbow dislocation associated with comminuted fractures of the medial humeral epicondyle and trochlea. The injury was managed conservatively with excellent results at the one‑year follow‑up. This report underscores that even complex injury patterns can achieve good outcomes without surgery when joint congruence and stability are maintained.

## Introduction

Traumatic elbow dislocation in children is an uncommon injury, representing approximately 3-6% of all pediatric elbow injuries [1]. Around 64-75% of elbow dislocations are associated with peri-articular fractures; among these, medial epicondyle fractures are the most frequently encountered, while lateral epicondylar fractures are the second most common [2,3]. The coexistence of medial condyle and trochlear fractures with elbow dislocation is exceptionally rare. Limited literature exists on the combination of elbow dislocation with medial humeral condyle fractures and trochlear fractures in pediatric patients, a condition that is seldom observed in young children. Medial humeral epicondyle fractures represent approximately 10-20% of elbow injuries in children, and 30-60% of these cases are associated with elbow dislocation. These injuries most commonly occur between the ages of 11 and 12 years and are four times more common in boys [4]. The mechanism is usually a valgus force exerted on the elbow when it is extended or slightly flexed [5]. Trochlear fractures are exceedingly rare injuries, and their exact incidence has not been clearly established in the literature. Isolated fractures may result from either direct trauma or avulsion mechanisms and can also occur in association with medial epicondyle fractures and elbow dislocations. Traditional management with cast immobilization is increasingly being replaced by early fixation and mobilization. Relative indications for surgical fixation include ulnar nerve entrapment, gross elbow instability, and fractures in athletic or other patients who require high-demand upper extremity function. Absolute indications for surgical intervention include incarceration of the fracture fragment within the joint or open fractures. Radiographic assessment of these injuries, the true degree of displacement, and their management remain controversial [6]. In this report, we present the case of an 11-year-old boy who sustained an elbow dislocation associated with fractures of the medial humeral condyle and trochlea.

## Case presentation

**Figure 1:**
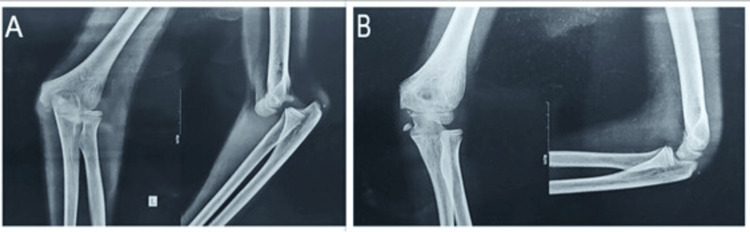
(A) Plain X-ray anteroposterior view and lateral view of the elbow joint after the injury. (B) Plain X-ray anteroposterior view and lateral view after closed reduction

**Figure 2:**
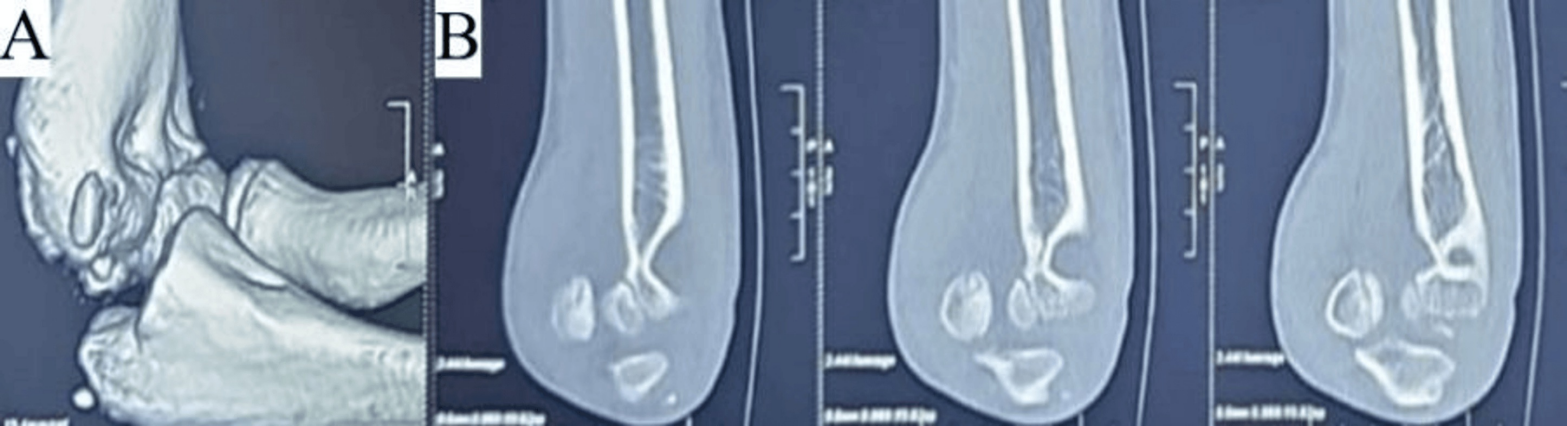
(A) Post-reduction CT of the elbow joint, 3D reconstruction view. (B) Sagittal view CT of the elbow joint CT, computed tomography

An 11-year-old boy sustained an injury to the left elbow following a fall from a bicycle and was taken to an outside hospital, where he was diagnosed with a left elbow dislocation and a medial humeral condyle fracture (Figure 1A). Closed reduction of the elbow was performed, and the joint was immobilized using an above-elbow plaster of Paris slab (Figure 1B). He was subsequently referred to our institution for further evaluation and management. On further physical examination, there were no signs of ulnar nerve entrapment. A CT scan of the left elbow (Figure 2) revealed a comminuted, displaced fracture involving the medial epicondyle and trochlea with intra-articular extension (Salter-Harris Type III), with the largest fracture fragment measuring 9.1 × 5.7 mm. At the time of presentation to our institution, the elbow was well reduced and stable. CT imaging demonstrated multiple small fracture fragments involving the medial epicondyle and trochlea, which were not amenable to fixation; therefore, the patient was managed non-operatively. The elbow was immobilized in a plaster of Paris slab for three weeks. Serial radiographs were obtained weekly during this period to ensure maintenance of ulnohumeral joint reduction. After three weeks of immobilization, the range of motion of the elbow was assessed periodically, and active mobilization was encouraged. Grip strength and flexor muscle strength were also evaluated. The range of motion of the elbow improved significantly throughout the follow-up period.

**Figure 3:**
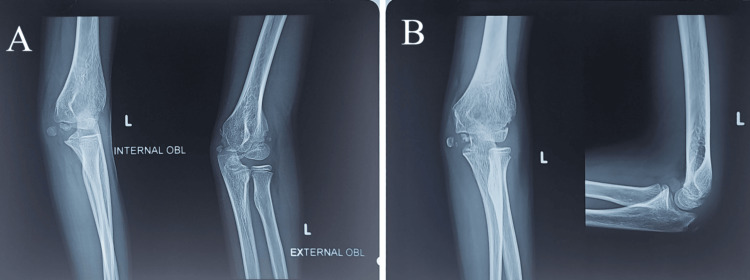
(A) Ninth-month follow-up X-ray showing internal and external oblique views. (B) AP and lateral views AP, anteroposterior

**Figure 4:**
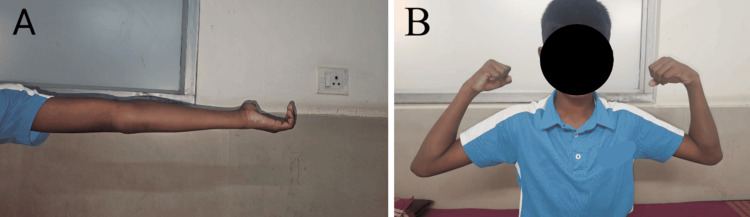
(A) Left elbow in full extension after the ninth-month follow-up. (B) Left elbow in flexion of 115 degrees and right elbow in full flexion

Subsequent follow-up radiographs (Figure 3) were obtained to assess the fracture fragments and joint space, which demonstrated signs of fracture healing. Periodic follow-up assessments were performed (Figure 4), and the range of motion of the elbow was evaluated, showing gradual improvement. Active mobilization of the elbow joint was encouraged.

## Discussion

Elbow dislocation associated with a medial humeral epicondyle fracture and a comminuted trochlear fracture is a rare occurrence in the pediatric population. Very few such cases have been reported in the English literature in children. Medial epicondylar fractures mainly occur through three mechanisms: direct trauma, avulsion injury, and elbow dislocation [6]. A direct blow to the posterior aspect of the medial epicondyle can cause a fragmented fracture associated with skin bruising. The most common mechanism is avulsion caused by sudden traction of the forearm flexor-pronator muscles, often associated with valgus stress, falls on an outstretched hand, or activities such as arm wrestling. In children, elbow extension during a fall increases valgus and muscular forces on the medial epicondyle, leading to avulsion. Another mechanism is elbow dislocation, especially posterolateral dislocation, in which the ulnar collateral ligament avulses the medial epicondyle; in 15-25% of cases, the fracture fragment may become trapped within the joint [7]. A pediatric trochlear fracture is a rare injury involving disruption of the structural integrity of the humeral trochlea within the elbow joint [8]. These fractures usually occur in association with injuries such as elbow dislocation, ligament damage, or fractures of the capitellum, radial head, or olecranon, rather than in isolation. Most cases result from significant direct trauma exceeding the strength of the bone and commonly require surgical management with open reduction and internal fixation. There is no standard pediatric classification system for trochlear fractures, and the number of children presenting with this fracture pattern is so small that an individual clinician is unlikely to gain sufficient experience in managing all such fracture patterns. Only a few articles have been published, and there is no clear protocol regarding the optimal management of these injuries. Since these fractures are exceedingly rare, treatment recommendations cannot be standardized because of the limited number of reported cases and clinical experiences. Management is based on fragment size, displacement, skeletal maturity, and patient activity [9]. Undisplaced or minimally displaced medial epicondyle fractures (<5mm) without ulnar nerve symptoms are generally managed conservatively using a posterior splint or above-elbow cast for a minimum of 3 to 4 weeks, which typically results in satisfactory healing and function. In unstable displaced medial epicondyle fractures with ulnar nerve symptoms, valgus laxity, and elbow instability, closed reduction and percutaneous K-wire fixation are feasible when the fracture fragment is amenable to fixation [10].

## Conclusions

Pediatric fractures involving the medial epicondyle and trochlea are uncommon elbow injuries that usually occur following trauma, frequently in association with elbow dislocation. Early recognition is essential because these injuries may be easily missed on initial radiographs due to the complex ossification pattern of the pediatric elbow. Careful clinical examination combined with appropriate imaging helps in accurate diagnosis and treatment planning. Management depends on the degree of displacement, elbow stability, associated intra-articular entrapment, and neurovascular status. Surgical intervention with open reduction and internal fixation is often required in displaced fractures or when associated with elbow instability, and incarcerated fragments are present. Timely anatomical reduction and stable fixation allow early mobilization and help restore joint congruity. This case highlights the importance of maintaining a high index of suspicion for combined medial epicondyle and trochlear fractures in children presenting with elbow trauma. Prompt diagnosis and appropriate management can lead to excellent functional outcomes with restoration of elbow movement and prevention of long-term complications such as stiffness, nonunion, instability, and growth disturbances.
